# Supplementary material for: Spatial and Temporal Volatility of PM2.5, PM10 and PM10-Bound B[a]P Concentrations and Assessment of the Exposure of the Population of Silesia in 2018–2021
Source: Int J Environ Res Public Health. 2022 Dec 22;20(1):138. doi: 10.3390/ijerph20010138 (PMC9819630; doi:10.3390/ijerph20010138)
Supplement: Supplementary file 1 [file ijerph-20-00138-s001.zip › ijerph-2085322-supplementary.pdf]

International Journal of
Environmental Research and Public Health

MDPI

Supplementary

Spatial and temporal volatility of PM2.5, PM10, and PM10-bound B[a]P concentrations and assessment of the exposure of the population of Silesia in 2018-2021

Dorota Kaleta and Barbara Kozielska*

Department of Air Protection, Silesian University of Technology, 22B Konarskiego St., 44-100 Gliwice, Poland
* Correspondence: barbara.kozielska@polsl.pl; Tel.: +48-32-237-15-30

Table S1. Detailed information on the location of selected urban background air quality monitoring stations in the Silesian Voivodeship.

No.	Measurement stations			PM2.5	PM10	B[a]P
1.	Bielsko-Biała	19 Kossak-Szczuckiej St.	49°48'48.5"N 19°01'38.3"E	+*)	+	+
2.	Częstochowa	2 Baczyńskiego St.	50°50'11.0"N 19°07'48.4"E	+*)	+	+
3.	Dabrowa Górnicza	25a Tysiąclecia St.	50°19'44.8"N 19°13'52.4"E		+	+
4.	Godów	Gliniki St.	49°55'18.8"N 18°28'16.6"E	+	+	+
5.	Katowice	6 Kossutha St.	50°15'52.6"N 18°58'30.1"E	+	+	+
6.	Knurów	5 Jedności Narodowej St.	50°13'59.4"N 18°39'20.6"E		+	+
7.	Myszków	3 Miedziana St.	50°34'47.0"N 19°19'36.1"E		+	+
8.	Pszczyna	Bogedaina St.	49°58'19.8"N 18°56'50.0"E		+	+
9.	Rybnik	37d Borki St.	50°06'40.3"N 18°30'58.1"E		+	+
10.	Tarnowskie Góry	Litewska St.	50°26'41.1"N 18°49'46.7"E	+	+	+

11.	Żywiec	83a Kopernika St.	49°40'17.8"N<br>19°14'04.0"E	+	+

*) from 2021

Table S2. Descriptive statistics of concentrations of PM2.5, PM10 and PM-bound B[a]P in the period 2018-2021.

Measurement stations	N	Mean	SD	Median	Min	Max	Coefficient of variation, %
				**PM10, µg·m⁻³**			
				2018			
Bielsko Biała	346	34.35	23.29	26.15	7.80	200.60	67.79
Częstochowa	344	33.40	19.46	26.55	10.20	162.70	58.28
Dąbrowa Górnicza	346	41.83	26.25	34.45	9.10	196.60	62.75
Godów	336	45.58	40.10	28.50	6.30	242.60	87.99
Katowice	361	40.00	24.26	32.90	10.40	181.90	60.65
Knurów	357	42.00	30.05	31.80	9.40	210.30	71.54
Myszków	333	49.10	38.38	33.40	9.10	230.60	78.18
Pszczyna	362	54.88	44.72	36.95	2.70	272.90	81.49
Rybnik	361	50.76	43.43	33.70	9.30	310.70	85.56
Tarnowskie Góry	365	38.82	24.80	29.90	7.80	168.90	63.88
Żywiec	335	44.33	36.50	29.80	8.20	240.70	82.34
				2019			
Bielsko Biała	349	27.54	19.88	20.40	4.00	216.00	72.18
Częstochowa	363	26.32	15.85	20.10	12.40	126.60	60.24
Dąbrowa Górnicza	351	32.91	22.26	25.90	8.80	130.90	67.63
Godów	340	38.03	31.23	25.80	9.70	211.80	82.13
Katowice	356	33.00	19.58	26.40	12.10	162.90	59.33
Knurów	349	35.04	27.49	25.90	8.50	180.30	78.45
Myszków	274	40.26	34.31	28.30	9.60	248.10	85.22
Pszczyna	358	44.29	39.12	29.00	10.10	248.80	88.31
Rybnik	356	42.97	41.06	27.45	9.10	271.80	95.57
Tarnowskie Góry	359	33.96	23.14	26.10	14.40	211.00	68.16
Żywiec	341	32.57	23.49	23.00	8.30	129.40	72.12
				2020			
Bielsko Biała	144	27.22	20.18	20.00	7.00	148.00	74.17
Częstochowa	365	22.87	11.07	19.40	4.40	84.30	48.41
Dąbrowa Górnicza	357	29.01	16.34	24.20	6.60	94.90	56.33
Godów	310	31.19	23.78	24.20	6.50	146.80	76.23
Katowice	365	28.74	14.15	24.90	9.40	112.80	49.22
Knurów	362	28.76	18.20	24.40	5.60	131.50	63.26
Myszków	315	35.28	24.17	27.50	7.70	155.30	68.49

Pszczyna	351	38.34	27.62	30.30	8.20	179.30	72.05
Rybnik	359	32.56	24.18	25.70	5.10	178.60	74.26
Tarnowskie Góry	358	28.77	15.50	24.00	4.20	104.40	53.87
Żywiec	365	33.34	29.08	23.00	6.90	295.80	87.22
2021							
Bielsko Biała	318	25.65	18.67	20.30	3.80	143.50	72.78
Częstochowa	362	26.08	19.89	20.20	2.20	170.30	76.26
Dąbrowa Górnicza	337	33.02	21.73	26.90	4.70	145.90	65.80
Godów	343	35.72	31.21	24.10	7.20	217.80	87.38
Katowice	347	26.76	22.33	18.40	3.50	158.20	83.44
Knurów	365	32.22	24.00	24.90	4.10	188.00	74.50
Myszków	276	36.97	28.51	27.10	5.90	166.40	77.12
Pszczyna	364	38.84	31.10	28.40	3.40	183.50	80.07
Rybnik	365	35.15	28.24	25.40	5.70	169.90	80.32
Tarnowskie Góry	365	27.96	19.40	22.50	3.90	149.00	69.40
Żywiec	364	37.10	30.59	26.35	6.00	173.70	82.45
**PM2.5, µg·m^{-3}**							
2018							
Bielsko Biała	0						
Częstochowa	0						
Godów	333	38.55	34.39	23.50	5.20	218.90	89.23
Katowice	339	29.99	21.35	22.10	7.90	142.40	71.18
Tarnowskie Góry	364	28.97	21.22	20.80	3.50	140.10	73.26
2019							
Bielsko Biała	0						
Częstochowa	0						
Godów	329	30.61	27.50	20.40	6.80	228.60	89.83
Katowice	358	24.10	17.22	18.20	6.50	126.10	71.47
Tarnowskie Góry	357	22.78	18.42	17.20	7.10	174.30	80.86
2020							
Bielsko Biała	0						
Częstochowa	0						
Godów	281	26.47	21.83	19.10	3.50	136.30	82.50
Katowice	365	20.19	11.52	16.30	5.70	84.90	57.07
Tarnowskie Góry	366	19.62	12.01	15.85	2.70	79.20	61.19
2021							
Bielsko Biała	365	20.80	19.11	14.10	1.00	134.40	91.85
Częstochowa	342	21.09	16.24	15.95	1.00	113.50	77.00
Godów	185	33.91	31.71	21.30	6.70	193.80	93.51
Katowice	361	22.65	17.11	17.10	3.50	132.20	75.51

Tarnowskie Góry	357	20.94	16.86	15.70	1.00	122.10	80.53
**PM-bound B[a]P, ng·m^{-3}**							
2018							
Bielsko Biała	351	4.47	5.47	2.31	0.15	20.13	122.48
Częstochowa	347	3.02	2.95	2.03	0.02	9.70	97.88
Dąbrowa Górnicza	181	5.12	5.55	2.98	0.11	16.00	108.33
Godów	340	9.26	11.83	4.14	0.17	54.18	127.69
Katowice	365	4.73	4.87	3.29	0.19	16.55	103.09
Knurów	181	6.92	7.00	3.84	0.26	20.83	101.24
Myszków	0						
Pszczyna	179	9.07	10.02	3.84	0.34	34.24	110.52
Rybnik	365	13.20	14.07	9.74	0.31	49.71	106.61
Tarnowskie Góry	183	4.40	4.47	2.85	0.14	14.63	101.61
Żywiec	344	7.99	8.88	5.51	0.20	40.13	111.08
2019							
Bielsko Biała	349	3.51	3.88	1.51	0.14	17.72	110.32
Częstochowa	365	2.69	2.76	1.41	0.12	10.90	102.49
Dąbrowa Górnicza	179	4.38	4.74	3.47	0.16	17.68	108.03
Godów	351	8.31	9.28	4.54	0.10	33.77	111.70
Katowice	362	4.06	4.23	2.35	0.16	14.25	104.37
Knurów	178	7.02	8.32	3.67	0.23	31.22	118.52
Myszków	180	7.79	8.32	5.97	0.20	33.14	106.71
Pszczyna	170	7.12	7.58	4.67	0.25	25.53	106.41
Rybnik	361	13.18	15.50	7.61	0.16	62.32	117.57
Tarnowskie Góry	180	3.92	4.88	2.29	0.12	21.46	124.53
Żywiec	346	6.10	6.21	3.49	0.18	20.79	101.74
2020							
Bielsko Biała	146	4.69	5.45	3.06	0.13	18.83	116.11
Częstochowa	365	2.48	2.21	2.24	0.10	10.48	89.09
Dąbrowa Górnicza	184	4.03	3.94	2.85	0.18	13.58	97.83
Godów	321	7.78	7.88	5.73	0.26	31.54	101.30
Katowice	366	3.49	3.07	2.70	0.23	12.60	88.08
Knurów	180	4.89	4.22	2.91	0.18	14.07	86.18
Myszków	162	8.84	6.17	9.89	0.43	22.41	69.71
Pszczyna	176	6.36	6.84	4.88	0.26	24.44	107.55
Rybnik	364	9.42	9.14	7.78	0.38	42.92	97.08
Tarnowskie Góry	183	4.39	4.58	2.85	0.21	20.69	104.15
Żywiec	366	7.82	8.79	4.52	0.24	36.49	112.36
2021							
Bielsko Biała	333	3.75	4.11	2.39	0.07	15.81	109.51

Częstochowa	365	2.93	2.92	2.23	0.11	14.05	99.61
Dąbrowa Górnicza	183	3.83	3.98	3.01	0.18	16.43	104.10
Godów	357	8.91	9.27	4.14	0.16	30.46	104.06
Katowice	353	3.64	3.84	2.02	0.04	13.74	105.48
Knurów	182	6.12	5.99	4.65	0.22	19.64	97.96
Myszków	174	8.82	7.83	7.84	0.18	25.88	88.77
Pszczyna	183	5.98	6.47	3.48	0.25	24.28	108.05
Rybnik	365	10.10	10.46	5.96	0.17	42.92	103.58
Tarnowskie Góry	183	3.43	3.82	2.08	0.15	20.69	111.31
Żywiec	364	8.35	8.86	5.61	0.16	29.63	106.10

stations for which 80% of the measurements were not covered during each year are marked in red

Table S3. Number of days exceeding the values of the daily mean concentrations of PM10 in the heating season and during the year.

	**2018**	**2019**	**2020**	**2021**	**2018**	**2019**	**2020**	**2021**
	acc. to Polish regulations				acc. toWHO regulations			
**Bielsko Biała**	52*	29	17	33	63	35	19	45
	52**	30	17	33	63	40	19	45
**Częstochowa**	52	25	13	30	69	35	20	40
	52	28	13	30	72	39	20	40
**Dąbrowa Górnicza**	80	47	40	52	98	55	48	59
	87	52	40	53	113	69	49	65
**Godów**	93	67	45	70	108	81	51	81
	94	79	46	70	109	96	52	82
**Katowice**	79	53	26	38	89	64	46	52
	81	57	26	38	93	72	48	52
**Knurów**	93	57	30	63	105	68	40	74
	93	63	30	63	108	79	42	75
**Myszków**	105	50	56	63	111	59	69	71
	106	63	59	63	115	74	72	71
**Pszczyna**	121	88	56	82	133	95	71	91
	126	107	77	83	139	116	96	94
**Rybnik**	113	75	48	73	122	84	59	82
	113	88	51	73	125	106	64	82
**Tarnowskie Góry**	78	53	30	39	100	65	39	51
	78	58	30	39	102	74	39	51
**Żywiec**	88	59	66	82	97	65	79	92
	88	63	66	83	97	71	80	93

where: $\frac{*}{**} = \frac{number\ of\ days\ in\ the\ heating\ season}{number\ of\ days\ in\ a\ year}$

Table S4. Number of days exceeding the values of the daily mean concentrations of PM2.5 in the heating season and during the year acc. to WHO regulations.

	2018	2019	2020	2021
**Bielsko Biała**	-	-	-	150
	-	-	-	173
**Częstochowa**	-	-	-	139
	-	-	-	186
**Godów**	176	124	119	106
	250	215	179	132
**Katowice**	166	132	140	171
	280	230	202	213
**Tarnowskie Góry**	176	123	141	149
	281	213	199	193
